# The nucleosome unwrapping free energy landscape defines distinct regions of transcription factor accessibility and kinetics

**DOI:** 10.1093/nar/gkac1267

**Published:** 2023-01-23

**Authors:** Benjamin T Donovan, Yi Luo, Zhiyuan Meng, Michael G Poirier

**Affiliations:** Biophysics Graduate Program, The Ohio State University, Columbus, OH 43210, USA; Biophysics Graduate Program, The Ohio State University, Columbus, OH 43210, USA; Biophysics Graduate Program, The Ohio State University, Columbus, OH 43210, USA; Biophysics Graduate Program, The Ohio State University, Columbus, OH 43210, USA; Department of Physics, The Ohio State University, Columbus, OH 43210, USA; Department of Chemistry & Biochemistry, The Ohio State University, Columbus, OH 43214, USA

## Abstract

Transcription factors (TF) require access to target sites within nucleosomes to initiate transcription. The target site position within the nucleosome significantly influences TF occupancy, but how is not quantitatively understood. Using ensemble and single-molecule fluorescence measurements, we investigated the targeting and occupancy of the transcription factor, Gal4, at different positions within the nucleosome. We observe a dramatic decrease in TF occupancy to sites extending past 30 base pairs (bp) into the nucleosome which cannot be explained by changes in the TF dissociation rate or binding site orientation. Instead, the nucleosome unwrapping free energy landscape is the primary determinant of Gal4 occupancy by reducing the Gal4 binding rate. The unwrapping free energy landscape defines two distinct regions of accessibility and kinetics with a boundary at 30 bp into the nucleosome where the inner region is over 100-fold less accessible. The Gal4 binding rate in the inner region no longer depends on its concentration because it is limited by the nucleosome unwrapping rate, while the frequency of nucleosome rewrapping decreases because Gal4 exchanges multiple times before the nucleosome rewraps. Our findings highlight the importance of the nucleosome unwrapping free energy landscape on TF occupancy and dynamics that ultimately influences transcription initiation.

## INTRODUCTION

Transcription factors (TFs) function by binding specific DNA sequences in gene promoters and enhancers to facilitate the recruitment of co-activators that in turn help initiate transcription (1), which typically occurs in bursts (2–5). In eukaryotes, TFs must target their binding sites in genomic DNA that is repeatedly wrapped in nucleosomes, the fundamental organizing unit of all eukaryotic genomes. The nucleosome contains ∼146 bp of DNA wrapped 1.65 times around a histone octamer core that contains two copies of the four core histones: H2A, H2B, H3 and H4 (6,7). While TF binding sites are often between nucleosomes in linker DNA where accessibility is regulated by higher order chromatin compaction (8–10), TF binding sites are also frequently found within the nucleosome near the DNA entry–exit region (11). The fully wrapped nucleosome sterically occludes most TFs because their DNA binding sites are wrapped around the histone octamer. However, nucleosomal DNA spontaneously unwraps, transiently exposing TF binding sites within the nucleosome, which is referred to as site exposure (12).

Nucleosome partial unwrapping is a key property that has been characterized by a range of methods including restriction enzyme accessibility (12,13), steady state Förster Resonance Energy Transfer (FRET) (14), stopped flow FRET (11,15), Fluorescence Correlation Spectroscopy (FCS) (15,16), small angle X-ray scattering (SAXS) (17,18), single-molecule force spectroscopy (19–21) and single-molecule FRET (smFRET) (22). Restriction enzyme accessibility measurements first showed that the probability of TF binding within partially unwrapped nucleosomes roughly decreases exponentially as the binding moves further into the nucleosome (12). Stopped flow measurements (16) have revealed that TF binding rates are reduced as their target site is moved further into the nucleosome, which implies that the site exposure probability is reduced. Additionally, nucleosomes accelerate TFs off their target site, further suppressing TF occupancy (22).

A complete characterization of nucleosome partial unwrapping requires the nucleosome unwrapping free energy landscape to be determined (23–26). This is a challenging landscape to measure because the relative probability of each unwrapped state needs to be determined. The measurement that most directly investigated this was reported by the Wang lab where they used single-molecule force spectroscopy to unzip the DNA extending out from the nucleosomes (23). As the junction between the single strand (ss) and double strand (ds) DNA propagates into the nucleosome and encounters DNA–histone interactions, the strand separation dwell time at base pairs adjacent to DNA-histone interactions change because the DNA needs to unwrap before it can unzip. From these measurements, a free energy landscape was inferred (24). This free energy landscape has been used to successfully model separately the impact of histone PTMs and DNA origami nanocalipers on nucleosome unwrapping (19,25).

While the nucleosome unwrapping free energy landscape is a key component for TF binding within partially unwrapped nucleosomes, other factors will contribute including: (i) the orientation of the binding site relative to the histone octamer since a 5 base pair change in position will rotate the orientation by 180°, (ii) the DNA footprint of the TF, (iii) the extent by which the TF wraps around the DNA and (iv) the size and orientation of TF regions that extend away from the DNA. Each of these factors could impact the extent the DNA must unwrap so that the TF does not sterically clash with the histone octamer or the adjacent nucleosomal DNA that remains wrapped around the octamer. These factors are related to the structure of the TF when bound to dsDNA and could vary significantly between different TF structural families. How the nucleosome unwrapping free energy landscape combines with these and other factors to influence TF occupancy with partially unwrapped nucleosomes is not well understood.

**Figure 1.**
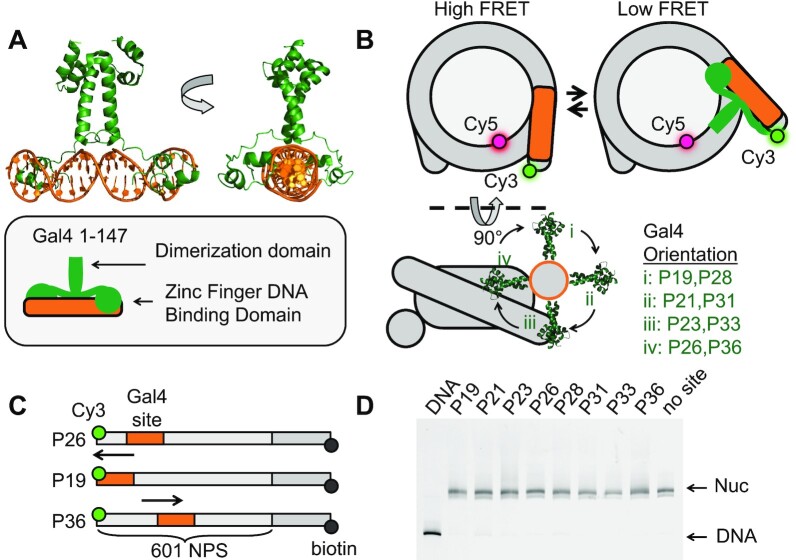
Experimental design to probe Gal4 binding throughout nucleosome entry-exit region. (**A**) Crystal structure of the transcription factor (TF) Gal4 bound to DNA as a dimer (PDB 3COQ) (28). The side view shows how Gal4 encompasses 270 degrees of DNA when bound. In this study, we are using amino acids 1–147 which includes the DNA binding domain and the dimerization domain. (**B**) Simplified cartoon of the nucleosome containing a FRET pair between Cy5-H2A(K119C) and Cy3-DNA. Gal4 binds by trapping the nucleosome in an unwrapped state which is detectable by FRET. Lower diagram illustrates the rotational position of Gal4 with respect to the octamer. This diagram illustrates how movement of the of the binding site by 5bp results in a 180-degree shift of the binding site with respect to the octamer. (**C**) Nomenclature used to refer to binding site position (i.e. P26 means binding site ends 26 bp from DNA edge). All binding sites were inserted into the Widom 601 nucleosome positioning sequence (NPS) (27). Additionally, the DNA contains a 75 bp linker terminated with a biotin required for surface tethering in single-molecule experiments. (**D**) All nucleosomes used in this study have the same mobility through a poly-acrylamide native gel indicating that insertion of the Gal4 binding site does not influence nucleosome positioning.

To understand TF occupancy within nucleosomes, we decided to focus on the well-studied budding yeast TF, Gal4, and investigate the equilibrium occupancy and the binding/dissociation kinetics at different positions within the nucleosome. Gal4 binds as a dimer to a quasi-palindromic 19 bp target sequence. The zinc finger binding domain of each monomer makes sequence specific interactions in the CGG major groove that are located at base pairs 2 through 4 at both 5 prime ends of the binding site. The two CGGs are oriented on opposite faces of the dsDNA resulting in Gal4 wrapping about 270° around the binding site (Figure 1A). The dimerization domain is orthogonal to the dsDNA helical axis and extends away from the center of the binding site. Unstructured regions of Gal4 connect each zinc finger to the dimerization domain, which likely results in Gal4 fluctuating between fully bound and partially bound states. We previously studied Gal4 binding to nucleosomes with its site located at the 8th to the 26th base pairs of the nucleosome containing the Widom 601 nucleosome positioning sequence (NPS) (27). At this location, the Gal4 dimerization domain is oriented toward the histone octamer (Figure 1B) and likely requires a significant amount of DNA unwrapping to bind within a partially unwrapped nucleosome.

Here, we used biochemical and FRET studies to investigate how Gal4 binding equilibrium and kinetics depend on target site position and orientation within the nucleosome. We found that nucleosome accelerated dissociation and Gal4 binding site orientation do not significantly contribute to the dependence of Gal4 occupancy on the target site position. Instead, it is primarily determined by the nucleosome unwrapping free energy landscape, where the Gal4 effective binding rate depends on its target site position within the nucleosome. Importantly, the unwrapping free energy landscape defined two distinct regions of Gal4 occupancy (inner and outer regions) that are separated by a sharp boundary at 30 bp into the nucleosome. Gal4 occupancy occurs in the nM concentration regime for binding sites that are completely in the outer region extending <30 bp into the nucleosome, while binding sites that extend into the inner region of the nucleosome require concentrations in the hundreds of nanomolar regime. Interestingly, the Gal4 binding/dissociation kinetics and the nucleosome unwrapping/rewrapping kinetics have very different dependencies on Gal4 concentration in these two regions. In the outer region, the effective binding rate of Gal4 is limited by the unwrapping/rewrapping equilibrium where the frequency of nucleosome unwrapping events that are trapped by Gal4 binding depends linearly on Gal4 concentration. In addition, the nucleosome rewrapping rate is independent of the Gal4 concentration where each time Gal4 dissociates the nucleosome rewraps. In contrast, in the inner region, the unwrapping rate limits the effective rate of Gal4 binding so that it becomes independent of Gal4 concentration even before the site is saturated. Furthermore, in this region, the nucleosome rewrapping frequency reduces as the Gal4 concentration increases because Gal4 dissociates and rebinds multiple times before the nucleosome rewraps. This implies that Gal4 significantly increases the time the nucleosome is in an unwrapped state. Overall, these findings highlight the complicated nature of TF binding within nucleosomes and how the nucleosome unwrapping free energy landscape is a central property of the nucleosome that impacts how TFs function within eukaryotes to regulate transcription initiation.

## MATERIALS AND METHODS

### Preparation of DNA molecules

Oligonucleotides (Supplementary Table S1) were ordered with a 5′ amino group modification or an amine modified thymine (Sigma Aldrich), labeled with Cy3 NHS ester (GE Healthcare), and purified by HPLC with 218TP C18 column (Grace/vydac). For the Cy3 location within the middle of the Gal4 target site (CCGGAGGGCTGCCCTCCGG), we used the central thymine to minimize the impact on Gal4 binding since this positions the Cy3 fluorophore opposite to the side of the DNA where Gal4 binds (28). Two types of DNA molecules were fabricated for these experiments: (i) a 147 bp nucleosome positioning sequence (NPS) consisting of the Widom 601 sequence (27) or (ii) the 601 NPS plus a 75 bp linker on the opposite side of the DNA from the binding site which is used to tether nucleosomes to a microscope slide for single-molecule measurements. The DNA was prepared by PCR with Cy3 fwd primer and biotin-labeled oligonucleotides from a plasmid containing the 601 NPS with the Gal4 2C sequence (29). Following PCR amplification, DNA molecules were purified using a MonoQ column (GE Healthcare).

### Preparation of histone octamers

Human recombinant histones were expressed and purified as previously described (30). Expression vectors were generous gifts from Dr Karolin Luger (University of Colorado) and Dr. Jonathan Widom. Mutations H3(C110A), H2A(K119C) H2A(E64C), and H2A(K15C) were introduced by site-directed mutagenesis (agilent). The histone octamer was refolded by adding each of the histones together with H2A and H2B in 10% excess of H3 and H4 and purifying as previously described (30). The refolded histone octamer was labeled specifically at either H2A(K119C), H2A(E64C), or H2A(K15C) with Cy5-maleamide (GE Healthcare) as previously described (31).

### Preparation of nucleosomes

Nucleosomes were reconstituted from Cy3-labeled DNA and purified Cy5-labeled histone octamer (HO) by double salt dialysis as previously described (31). Dialyzed nucleosomes were loaded onto 5–30% sucrose gradients and purified by centrifugation on an Optima L-90 K Ultracentrifuge (Beckman Coulter) with a SW-41 rotor. Sucrose fractions containing nucleosomes were collected, concentrated, and stored in 0.5× TE pH 8 on ice.

### Preparation of gal4

To prepare Gal4, amino acids 1–147 of Gal4 were expressed from plasmid pET28a containing Gal4-1–147 in E. coli Rosetta (DE3)pLysS cells (Millipore, 70956) by inducing with 1 mM IPTG + 10 μM ZnAc for 3 h. Cells were harvested by centrifugation and resuspended at 50 ml per 1 l starting culture in buffer A (50 mM Tris pH 8.0, 200 mM NaCl, 1 mM DTT, 10 μM ZnAc, 1 mM phenylmethanesulfonyl fluoride (PMSF), 20 μg/ml leupeptin and 20 μg/ml pepstatin) and stored at –80°C. The cells were lysed by sonication and clarified by centrifugation, loaded onto a 5 ml HisTrap HP Ni-NTA column (GE Healthcare, 17524801), equilibrated with buffer B (25 mM Tris pH 7.5, 200 mM NaCl, 0.2% Tween-20, 10 mM imidazole, 20 μM ZnAc, 1 mM DTT, 1 mM PMSF), and eluted with elution buffer (25 mM Tris pH 7.5, 200 mM NaCl, 0.2% Tween-20, 200 mM imidazole, 20 μM ZnAc, 1 mM DTT, 1 mM PMSF). Fractions containing Gal4 were dialyzed into buffer C (25 mM Tris pH 7.5 200 mM NaCl, 20 μM ZnAc, 1 mM DTT, 1 mM PMSF) and purified with a TSKgel SP-5PW cation exchange column (Tosoh, 07161). Fractions containing Gal4 were concentrated using Amicon 10K filters (Millipore, UFC201024) and stored in buffer D (HEPES pH 7.5, 200 mM NaCl, 10% glycerol, 20 μM ZnAc, 1 mM DTT, 1 mM PMSF).

### Ensemble FRET measurements

Gal4 binding to Cy3–Cy5 nucleosomes was measured as previously described (14,22). 0.2 or 0.5 nM nucleosomes were incubated for at least 5 minutes with 0–3000 nM Gal4 in 10 mM Tris–HCl pH 8, 130 mM NaCl, 10% glycerol, 0.0075% v/v Tween-20. Fluorescence emission spectra were acquired as previously described (22). FRET efficiency was measured using the (Ratio)_A_ method (32).

### Electrophoresis mobility shift assays

0.5 nM DNA or nucleosomes were incubated with TF in 10 mM Tris–HCl (pH 8), 130 mM NaCl, 10% glycerol, 0.0075% v/v Tween-20 for at least 5 min and then resolved by electrophoretic mobility shift assay (EMSA) with a 5% native polyacrylamide gel in 3× TBE.

### Single-molecule TIRF microscope

The smTIRF microscope was built on an inverted IX71-inverted microscope (Olympus) as previously described (33). 532 and 638 nm diode lasers (Crystal Lasers) were used for Cy3 and Cy5 excitation, respectively. The excitation beams were expanded and then focused through a quartz prism (Melles Griot) at the surface of the quartz flow cell. A 1.35 N.A. water-immersion objective (Olympus) was used to collect fluorescence. Images of the Cy3 and Cy5 fluorescence were then split into separate images using a DualView system (Optical Insights) with a dichroic mirror (Chroma Technology, T635lpxr), and two band-pass filters with 30 nm bandwidth centered at 585 nm (Chroma Tech., D585/30) and at 680 nm (Chroma Tech., D680/35) for the Cy3 and Cy5 channels, respectively. The images from the two fluorophores were aligned side by side so that each of them occupies one half of the surface area on the CCD chip in a PhotonMax EMCCD camera (Princeton Instruments). Each video was acquired using WinView software (Roper Scientific).

### Flow cell preparation

Quartz microscope slides (G. Finkenbeiner) were functionalized with poly-ethylene glycol (PEG, Laysan Bio, MPEG-SVA-5000) and biotin-PEG (Laysan Bio, Biotin-PEG-SVA-5000) and assembled with glass cover-slips to make the flow cell. Quartz microscope slides and glass coverslips were cleaned in toluene and ethanol with sonication, and then further cleaned in Piranha solution (3:1 mixture of concentrated sulfuric acid to 50% hydrogen peroxide) and washed in 1M sodium hydroxide. The cleaned slides were treated with 2% v/v 3-aminopropyl-triethoxysilane (MP biomedicals 215476680) in acetone, and then with 10% w/v PEG in 0.1M potassium tetraborate pH 8.1 (100:1 mass ratio mixture of mono-functional PEG to biotin-PEG). Functionalized quartz slides and coverslips were adhered using parafilm with rectangular regions removed to define flow cells. Before each experiment, the flow cell is treated sequentially with 1 mg/ml BSA, 20 mg/ml streptavidin and biotin-labeled DNA/nucleosome samples to form surface tethers.

### Single-molecule fluorescence measurements of gal4 binding kinetics

Biotinylated nucleosomes were allowed to incubate in the flow cell at room temperature for 5 min and then washed out with imaging buffer containing the desired concentration of Gal4. The samples were first exposed to 638 nm excitation to determine the location of Cy5-labeled molecules and then 532 nm for FRET measurements. The imaging buffer for FRET experiments contained 10 mM Tris–HCl pH 8, 130 mM NaCl, 10% glycerol, 0.5% v/v Tween-20, 0.1 mg/ml BSA, 2 mM Trolox, 0.0115% v/v COT, 0.012% v/v NBA, 450 μg/ml glucose oxidase (Sigma G2133) and 22 μg/ml catalase (Sigma C3155).

Single-molecule time series were fit to a two-state step function by the hidden Markov method using vbFRET (34). Evidence of multiple low FRET states in some traces were observed. However, the FRET traces were analyzed as a single low FRET state because it was unclear the number of low FRET states and the two FRET state model captured all the key trends. We speculate the small differences in FRET values are related to small changes in position of the Gal4 binding site because of shifts in the histone octamer position (20) and changes in nucleosomal DNA stretching (35), and variations in Gal4 orientation relative to the histone octamer when bound to its target site within partially unwrapped nucleosome. Idealized time series were further analyzed using custom written Matlab programs to determine the dwell-time distributions of the TF bound and unbound states. Dwell-time and unbound-time cumulative sum distributions were generated from these traces and each distribution was analyzed using MEMLET to determine the best fit for the data and ultimately obtain rate constants for the transitions between bound and unbound states (36).

### Three state model that describes the dependence of single-molecule FRET fluctuations on gal4 concentration

The Gal4 concentration dependence of the nucleosome unwrapping and rewrapping kinetics was determined from a three state model where: N_1_ is the probability of the wrapped nucleosome states that cannot be bound by Gal4, N_2_ is the probability of the unwrapped nucleosome states that can be bound by Gal4, and N_3_ is the probability of the unwrapped nucleosome states that are bound by Gal4. To describe the kinetics for the transition into state N_3_, we set *k*_32_ to zero and used the rate matrix:

$$\begin{equation*}R = \left( {\begin{array}{@{}*{3}{c}@{}} { - {k_{12}}} & \quad {{k_{21}}} & \quad 0\\ {{k_{12}}} & \quad { - {k_{21}} - {k_{23}}\left[ {Gal4} \right]} & \quad 0\\ 0 & \quad {{k_{23}}\left[ {Gal4} \right]} & \quad 0 \end{array}} \right) \end{equation*}$$

The master equation for the time evolution of the probability of the states is

$$\begin{equation*} \frac{d}{{dt}}X ({t} ) = R \cdot X, \ {\rm where} \ X = \left( {\begin{array}{@{}*{1}{c}@{}} {{N_1}( t )}\\ {{N_2}( t )}\\ {{N_3}( t )} \end{array}} \right). \end{equation*}$$

The solution to the master equation is:

$$\begin{equation*} X({\rm t}) = {C_0} {\rm exp}({\lambda_0} t) + {C_{\text{-}}} {\rm exp}({\lambda_{\text{-}}} t) + {C_{+}} {\rm exp}({\lambda_{+}} t) , \end{equation*}$$

where ${\rm{ }}{C_i} = \left( {\begin{array}{@{}*{1}{c}@{}} {{N_{i1}}}\\ {{N_{i2}}}\\ {{N_{i3}}} \end{array}} \right)$, (i = 0, -, +) are the eigenvectors of R and *λ_i_* are the eigenvalues of *R*. To simplify the solution to the master equation, we took advantage of two observations. (i) The nucleosome unwrapping rate *k_12_* is on the hertz scale, so it is always small relative to the rewrapping rate (12,16) (*k*_12_ << *k*_21_). (ii) For the Gal4 concentrations explored in this study, the Gal4 binding rate to a *fully exposed* naked DNA site is significantly larger than the nucleosome unwrapping rate (*k*_12_ << *k*_23_[Gal4]). The nucleosome site is exposed 1 percent of the time or less, so the measured binding rates inferred from unwrapping rates is more than 100-fold less than the Gal4 binding rate to naked DNA. For these conditions, the eigenvalues for *R*_unwrap_ are *λ*_0_ = 0, *λ_-_* = –*k*_21_ – *k*_23_ [Gal4], and *λ_+_* = –*k*_12_/(1 + *k*_21_*/*(*k_23_* [Gal4])). Also, it is a good approximation to assume that the system starts in state N_1_ since *k*_12_ << *k*_21_. So, the eigenvectors are ${\rm{\ }}{C_0} = \left( {\begin{array}{@{}*{1}{c}@{}} 0\\ 0\\ 1 \end{array}} \right)$, ${C_ - } = \left( {\begin{array}{@{}*{1}{c}@{}} 0\\ 0\\ 0 \end{array}} \right)$, and ${C_ + } = \left( {\begin{array}{@{}*{1}{c}@{}} 1\\ 0\\ { - 1} \end{array}} \right)$. This implies *N*_1_ = exp(*λ_+_ t*), N_2_ = 0, N_3_ = 1 – exp(*λ_+_ t*). Here, the rates out of state N_2_ are so fast that this state is essential never populated. Therefore, the transition rate from the high FRET state where the nucleosome is fully wrapping into the low FRET state where the nucleosome is partially unwrapped and bound by Gal4 depends on the Gal4 concentration as *k_H__→__L_* = *k*_12_/(1 + *k*_21_*/*(*k_23_* [Gal4])).

The rate matrix for determining the rate into the state *N*_1_ where the nucleosome is fully wrapped and not bound by Gal4 is:

$$\begin{equation*} R = \left( {\begin{array}{@{}*{3}{c}@{}} 0 & \quad {{k_{21}}} & \quad 0\\ 0 & \quad { - {k_{21}} - {k_{23}}[ {Gal4} ]} & \quad {{k_{32}}}\\ 0 & \quad {{k_{23}}[ {Gal4} ]} & \quad { - {k_{32}}} \end{array}} \right). \end{equation*}$$

Here, *k*_12_ is set to zero and *k*_32_ is non-zero. Again, to simplify the solution to the master equation, we took advantage of two observations. The Gal4 dissociation rate *k*_32_ is small relative to (i) the nucleosome rewrapping rate (*k*_32_ << *k*_21_) and (ii) the Gal4 binding rate at a fully exposed site for the Gal4 concentrations studied here (*k*_32_ << *k*_23_[Gal4]). We used the same approach as above to determine transition rate from the low FRET state into the high FRET state. This results in the low to high FRET transition rate to depend on the Gal4 concentration as *k*_L__→__H_ = *k*_32_/(1+ (*k*_23_ [Gal4])*/k*_21_). We then fit the rates as a function of [Gal4] with *k*_L__→__H_ and *k*_H__→__L_.

## RESULTS

### Gal4 has two binding modes at sites that extend up to 23 base pairs into the nucleosome

To investigate the position dependence of Gal4 binding within the nucleosomes, we prepared a set of eight different nucleosomes each with the Gal4 binding site at a different position relative to the nucleosome (Figure 1). We adjusted the binding site position in steps of 2–3 bp: (i) P19 (bp 1 to bp 19), (ii) P21 (bp 3 to bp 21), (iii) P23 (bp 5 to bp 23), (iv) P26 (bp 8 to bp 26), (v) P28 (bp 10 to bp28), (vi) P31 (bp 13 to bp 31), (vii) P33 (bp 15 to bp 33), (viii) P36 (bp 18 to bp 36). The DNA was 5′ labeled with Cy3 at the end nearest the Gal4 binding site, while Cy5 was attached to H2A(K119C). These binding sites scan over nearly two helical turns of the nucleosomal DNA allowing for high resolution mapping of the Gal4 occupancy as a function of binding site position within the nucleosome.

**Figure 2.**
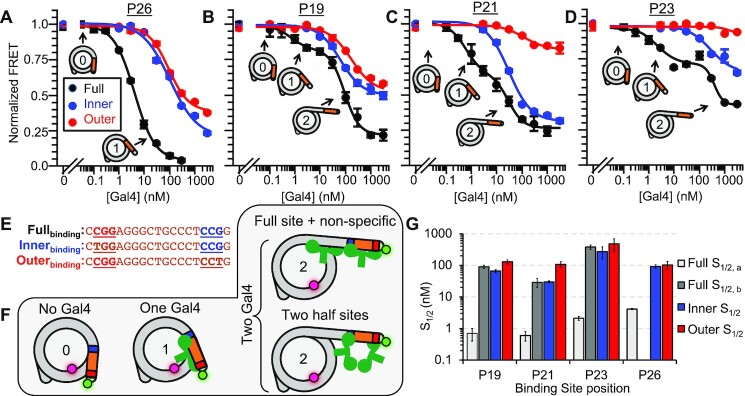
Gal4 binds with nanomolar affinity to Gal4 sites contained before the 30 bp barrier. (**A–D**) Ensemble FRET measurements of Gal4 binding to nucleosomes with a binding site at the position indicated above the plot. To the full binding site (black), the binding curves for P19, P21 and P23 exhibit two inflection points and are fit to the sum of two binding isotherms, while P26 is fit to a single binding isotherm. To the half site mutants, the FRET decrease begins at significantly higher concentrations and is fit to only one binding isotherm. Error bars indicate ±1 SEM. (**E**) Gal4 binding sites used in this study. In the ‘inner’ (blue) and ‘outer’ (red) DNA sequences, a ‘T’ is swapped into the 3 bp region recognized by the Zn-finger. This DNA mutation weakens the interaction of a Gal4 monomer with DNA. (**F**) A model describing multiple states of nucleosome unwrapping. State 1 represents Gal4 bound to its specific site within a nucleosome. This specific binding facilitates additional Gal4 binding and, subsequently, further nucleosome unwrapping. (**G**) Summary of *S*_1/2_ values from all fits. Error bars indicate ±1 standard error of the mean (SEM).

**Figure 3.**
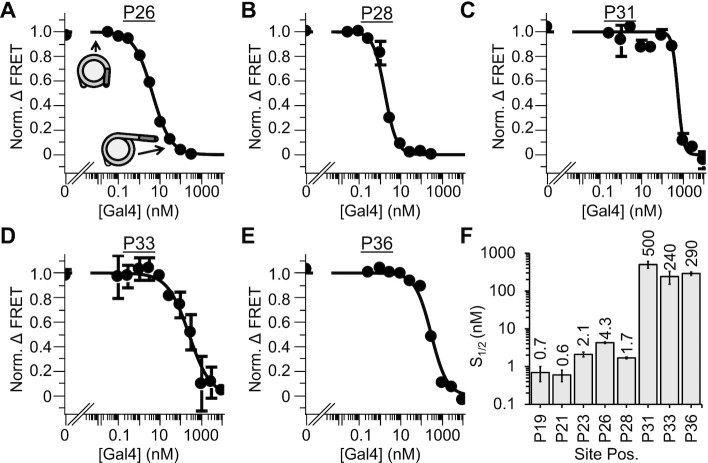
Gal4 binding is reduced ∼100-fold to binding sites extending past 30 bp into the nucleosome. (**A–E**) Ensemble FRET measurements of Gal4 binding to nucleosomes containing a ‘full’ Gal4 binding site at the indicated position. All binding curves are fit to a single binding isotherm. Error bars indicate ±1 SEM. (**F**) Summary of all *S*_1/2_ values from Figures 2 and 3. Error bars indicate ±1 SEM.

We carried out Gal4 titrations with each of these eight nucleosome constructs (Figures 2 and 3). As previously reported the Gal4 titration with P26 nucleosomes fits to a single binding isotherm with an *S*_1/2_ of 4.3 ± 0.2 nM (Figure 2A) (37). In contrast, the first 3 Gal4 positions at P19, P21 and P23 required a Gal4 titration that extended over nearly 4 orders of magnitude for the reduction in FRET to saturate (Figure 2B–D). Therefore, these three Gal4 titrations could not be fit with a single binding isotherm. Instead, these titrations required fits to the sum of two binding isotherms. These fits establish two separate binding S_1/2_ concentrations and imply that two Gal4 dimers bind the nucleosome where second binding contributes to further unwrapping (Supplementary Figure S1). To control for non-specific Gal4 binding to the nucleosome, we carried out Gal4 titrations with nucleosomes that did not contain a binding site (Supplementary Figure S2A). We observe at most a 10% reduction at about 1 uM of Gal4. Therefore, both binding events at P19, P21 and P23 appear to require the Gal4 binding site.

To understand how two Gal4 dimers bind the nucleosome with one Gal4 binding site, we considered two possible mechanisms. The first mechanism is that each Gal4 dimer binds only half of the target sequence, where the first Gal4 binds the outer half of its target site, while the second Gal4 binds in the inner half of its target site. The second mechanism we considered is where the first Gal4 dimer binds the full binding site, which then facilitates binding of the second Gal4 dimer. In this second mechanism, the final state with two Gal4s bound could be where either one Gal4 is bound to the full target site with the second non-specifically bound, or where the two Gal4 are each partially bound to the target site (Figure 2E-F). To differentiate between these two mechanisms, we took advantage of the 3 base pair sequence of the Gal4 binding site, CCG, which is located at the outside edges of the full binding site and directly interacts with the zinc finger DNA binding domain of each Gal4 monomer (Figure 1A). We prepared nucleosomes with the Gal4 binding site mutated so either the inner CCG (Inner_binding_) or outer CGG (Outer_binding_) of the full site is retained (Figure 2E, Supplementary Figure S3). These Inner_binding_ and Outer_binding_ sites result in the same reduced Gal4 binding affinity to its target site on DNA (Supplementary Figure S2B) by limiting Gal4 binding largely to one side of the target sequence (29). Therefore, if the first Gal4 binds nucleosomes via the half-site mechanism then the Outer_binding_ site should retain a similar *S*_1/2_ value to that of the Full_binding_ site. However, if both Inner_binding_ and Outer_binding_ mutations result in a significantly higher Gal4 *S*_1/2_, these results would indicate that the first Gal4 dimer binds the full target sequence within the nucleosome.

We carried out Gal4 titrations with nucleosomes containing either the Inner_binding_ or Outer_binding_ site at positions P19, P21, P24 and P26 (Figure 2A–D). We find that the Gal4 *S*_1/2_ for both the Inner_binding_ and Outer_binding_ sites at each position have a much higher S_1/2_ than the Full_binding_ Gal4 site. This implies that the lower *S*_1/2_ (higher affinity) for Gal4 binding to the full binding site at each position is due to site specific binding of one Gal4 dimer to the full target site (Figure 2F). Therefore, the smaller S_1/2_ of 0.7 ± 0.3 nM, 0.6 ± 0.2 nM, 2.1 ± 0.3 nM and 4.3 ± 0.2 nM represents the apparent dissociation constant for Gal4 binding specifically to its full site within P19, P21, P23 and P26 nucleosomes, respectively and is consistent with previous studies of dCas9 binding to sites within the first 25 base pairs of the nucleosome (38). In addition, these results indicate that the second Gal4, which binds with a larger *S*_1/2_ of 90 ± 10, 29 ± 9 and 380 ± 60 nM, is due to a second Gal4 binding event to P19, P21 and P23 nucleosomes, respectively. Importantly, second binding appears to be facilitated by the site-specific binding since Gal4 does not significantly reduce the FRET efficiency without the target sequence (Supplementary Figure S2A).

### Gal4 occupancy at nucleosomal sites changes by two orders of magnitude as the target site extends more than 30 base pairs into the nucleosome.

To quantify Gal4 occupancy at sites positioned further into the nucleosome, we carried out FRET efficiency measurements of Gal4 titrations to P29, P31, P33 and P36 nucleosomes and fit them to binding isotherms (Figure 3 A–E). Each Gal4 titration fits well to single binding isotherms with an *S*_1/2_ of 1.7 ± 0.1, 500 ± 100, 240 ± 90 and 290 ± 30 nM, respectively. The entire range of *S*_1/2_ values, which are also the apparent dissociation constants, are summarized in Figure 3F. These results reveal that for Gal4 binding sites that extend by less than 30 bp into the nucleosome, the apparent dissociation constant is in the nanomolar range, while for sites that extend >30 bp, the apparent dissociation constant is in the hundred nanomolar range. This implies there is an abrupt decrease in nucleosomal DNA accessibility that occurs for binding sites that extend more than 30 bp into the nucleosome, suggesting there is a ‘barrier’ at this location between accessible and inaccessible nucleosomal DNA.

The orientation of the Gal4 binding site relative to the histone octamer has a minor impact on Gal4 occupancy at its full site but significantly impacts the Gal4 binding mode at partial Gal4 binding sites within the nucleosome. The Gal4 binding site goes through nearly two full rotations as the site is shifted from P19 to P36. Therefore, the comparison of Gal4 occupancy at these different positions provides insight into whether binding site orientation influences Gal4 occupancy. For example, if the binding site orientation was the primary factor that influenced Gal4 occupancy within the nucleosome then the Gal4 *S*_1/2_ would return close to its original value after a shift of 10 bp. As the binding site is shifted from P19 to P28, the *S*_1/2_ increases from 0.6 to 4.3 nM and back down to 1.7 nM. This oscillation in the *S*_1/2_ suggests that in the first 30 base pairs of the nucleosome, binding site orientation influences Gal4 occupancy (Figure 3F). However, the single abrupt increase in the *S*_1/2_ as the binding site extends more than 30 bp into the nucleosome is not due to helical orientation but instead an abrupt reduction in the nucleosome unwrapping probability. Overall, these results indicate that the distance a TF binding site extends into the nucleosome has a much larger impact on TF occupancy than the binding site orientation.

The relative change in FRET efficiency induced by Gal4 titrations to nucleosomes with the Inner_binding_ or Outer_binding_ site mutations at the different binding site locations (P19, P21, P23, P26) provides additional insight into how the binding site orientation influences partially bound Gal4. The orientation of the binding sites relative to the nucleosome are rotated about 90 degrees for each location (Figure 1B). In addition, only one of the two zinc finger DNA binding domains of the Gal4 dimer will preferentially bind to the one CGG in the mutated target site. For P21 and P23, the outer CCG of the binding site is exposed by facing away from the histone octamer and the adjacent DNA gyre, while the inner CCG is sterically blocked because it is facing toward the histone octamer or the adjacent DNA gyre (Figure 1B). In contrast, for P19 and P26, the outer CCG is sterically blocked by facing toward the histone octamer or the adjacent DNA gyre, while the inner CCG is exposed by facing away from the histone octamer and adjacent DNA gyre.

The large reduction in FRET observed for Gal4 binding to both full and mutant sites at positions P19 and P26 implies significant and similar DNA unwrapping from the nucleosome (Figure 2C, F). In contrast, the relative change in FRET is significantly smaller for the Outer_binding_ site than the Inner_binding_ site at the P21 and P23 positions (Figure 2D, E). This implies that Gal4 binding to the Outer_binding_ site requires much less DNA unwrapping than binding to the Inner_binding_ site. Comparison of these results suggest that significant DNA unwrapping is required for partial Gal4 binding to the inner CGG irrespective of its orientation relative to the nucleosome. In contrast, the outer CGG requires significant DNA unwrapping only if it is oriented toward the histone octamer or the adjacent DNA gyre, while significantly less DNA unwrapping is required for partial Gal4 binding to the outer CGG if it is facing away from the histone octamer and adjacent DNA gyre. Overall, these results imply that the distance the site extends into the nucleosome largely dictates Gal4 occupancy at its full site while binding site orientation dictates the magnitude of unwrapping required for Gal4 to engage partial binding sites within the nucleosome.

### Gal4 binding does not require nucleosomal DNA to unwrap significantly past its DNA binding site

**Figure 4.**
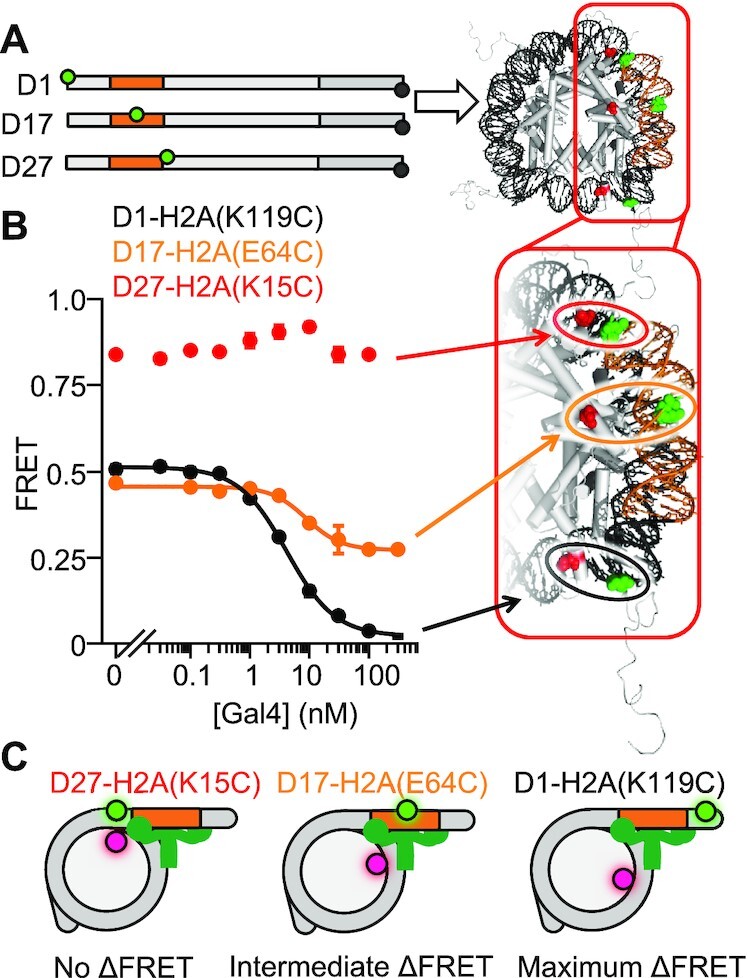
The nucleosome does not unwrap past the Gal4 binding site. (**A**) Schematic of DNAs containing Cy3 donor fluorophore either 5′ of the Gal4 binding site (D1), in the middle of the Gal4 binding site (D17) or 3′ of the Gal4 binding site (D27). These DNAs are reconstituted into nucleosomes containing a Cy5 acceptor fluorophore at H2A(K119C), H2A(E64C) or H2A(K15C) for D1, D17 or D27 respectively. (**B**) Ensemble FRET measurements of Gal4 binding to nucleosomes described in (A). (**C**) Summary of results: while we observe nucleosome unwrapping 5′ of the binding site and within the Gal4 binding site, we observe no nucleosome unwrapping for DNA beyond the Gal4 binding site.

The amount of DNA required to unwrap so that Gal4 can bind its target site could extend significantly beyond the target site because of the steric bulk of Gal4. To investigate the amount of DNA that is required to unwrap for Gal4 to bind, we carried out Gal4 binding titrations with the Cy3–Cy5 fluorophores inserted at different positions within the nucleosome (Figure 4A). The Cy3 and Cy5 fluorophore pair undergoes efficient Forster Resonance Energy Transfer (FRET) when they are within 1–3 nanometers of each other and decreases to about 50% at a distance of 5–6 nanometers. We attached the Cy3 (donor) fluorophore to the DNA and the Cy5 (acceptor) fluorophore to H2A so that unwrapping is detected (i) outside (Cy3 at bp 1 and Cy5 at H2A(K119C)), (ii) in the middle (Cy3 at bp 17 and Cy5 at H2A(E64C)), or (iii) inside (Cy3 at bp 27 and Cy5 at H2A(K15C)) the Gal4 recognition site at P26 (Figure 4A).

We carried out Gal4 titrations with each of the nucleosome constructs and determined the FRET efficiency at each concentration (Figure 4B) using the Ratio_A_ method (32). As discussed above and as previously reported (37), we detect over a 50% reduction in the FRET efficiency as Gal4 binds to its site within partially unwrapped nucleosomes containing the Cy3–Cy5 fluorophore pair outside the binding site with an *S*_1/2_ = 4.3 ± 0.2 nM. We then carried out Gal4 titrations with nucleosomes containing the Cy3–Cy5 at the middle position. This resulted in a decrease in the FRET efficiency, albeit with only a 20% reduction. Furthermore, the S_1/2_ for Gal4 binding to nucleosomes with the middle label position is 9 ± 1 nM, which is similar to the S_1/2_ for binding nucleosomes with the Cy3–Cy5 pair at the outer position. Finally, we carried out Gal4 titrations with Cy3–Cy5 at the inner position. At this position, there was no reduction in FRET over the concentration range that Gal4 binds to its site within partially unwrapped nucleosomes. Instead, there is about a 10% increase in the FRET. In combination, these results indicate that while Gal4 traps the nucleosome in a partially unwrapped state, (i) the outer portion of the nucleosomal DNA is shifted significantly away from the histone octamer, (ii) the middle portion of the Gal4 site is shifted away from the histone octamer but at a lesser extent than the outer portion, and (iii) the inner portion of the Gal4 binding site is not significantly unwrapped away from the octamer. Overall, these results indicate that Gal4 binding to its site within the nucleosome does not require the DNA to unwrap significantly past its binding site.

### The abrupt decrease in the Gal4 occupancy for target sites of >30 bp into the nucleosome is due to a decrease in the Gal4 binding rate.

The apparent dissociation constant (*K*_D_) of Gal4 occupancy at its site within the nucleosome is set by the ratio of the effective binding rate constant and the dissociation rate. Previously, it was demonstrated that the unwrapping/rewrapping equilibrium reduces the effective Gal4 binding rate constant (15,16). More recently, we showed that nucleosomes increase Gal4 dissociation rates from sites within the nucleosome, and this effect can even be larger than the change in the binding rate constant (22). Based on these published observations, we investigated if changes in the effective binding rate constant and/or the dissociation rate are responsible for the 2 orders of magnitude increase of the Gal4 *S*_1/2_ as the target site position is moved more than 30 base pairs into the nucleosome.

**Figure 5.**
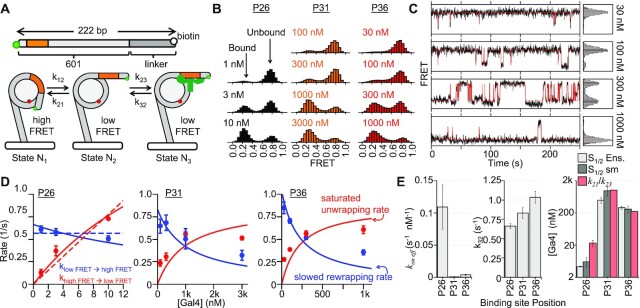
Single-molecule measurements of Gal4 binding throughout the entry–exit region. (**A**) Single-molecule measurements are performed on a 222 bp DNA molecule containing the Gal4 binding site at varied positions within the 601 NPS and a 75 bp biotinylated linker. Below: cartoon representation of the Polach and Widom site exposure model (12). For all single-molecule measurements, the FRET pair is between end-labeled Cy3-DNA and Cy5-H2A(K119C) as in Figures 2 and 3. (**B**) Histograms of nucleosome FRET efficiency at multiple concentrations of Gal4. For all binding site positions, increased Gal4 concentration promotes formation of the low FRET (unwrapped) state. (**C**) Representative time traces showing P36 nucleosome FRET fluctuations at 4 Gal4 concentrations. As Gal4 concentration increases, the nucleosome spends more time in the low FRET state. (**D**) High→ low (red) and low → high (blue) rates for Gal4 binding to P26, P31, and P36 nucleosomes. Solid lines represent fits to 3 state binding model depicted in Figure 5A. The dashed, linear fit for P26 represents a 2-state model where binding rate (red) increases linearly with concentration and dissociation rate does not depend on concentration. It is not possible to fit P31 and P36 to the 2-state model. Error bars indicate ±1 standard deviation (SD). (**E**) Summary of data from single-molecule measurements, *left: k_on eff_*, *middle:* the Gal4 dissociation rate (*k*_32_), and *right*: *S*_1/2_ values from both single-molecule FRET and ensemble FRET measurements (light gray and dark gray, respectively). This plot also includes *k*_21_/*k*_23_ (in red), the concentration at which the Gal4 binding rate (*k*_23_) exceeds the Gal4 rewrapping rate (*k*_21_). Error bars indicate ±1 SD.

To investigate this, we carried out smFRET measurements to determine the Gal4 binding rate constants and dissociation rates at different binding site positions within the nucleosomes. We decided to focus on binding to P26, P31 and P36 to determine the impact of Gal4 binding site position on Gal4 kinetics. For the experiments, a biotin molecule that is attached at the 5′ end of the DNA, which is opposite to the Gal4 binding site (Figure 1B), is used for tethering to a PEG coated quartz slide surface with streptavidin (Figure 5A). We acquired time traces of Cy3 and Cy5 fluorescence from over 400 individual nucleosomes at each binding site position and for three to four separate Gal4 concentrations (Figure 5B, Supplementary Figure S4). By monitoring FRET efficiency, we observe that the nucleosomes fluctuate between high (fully wrapped) and low (partially unwrapped) FRET efficiency nucleosome states. These FRET state transitions provide information on nucleosome unwrapping/rewrapping and Gal4 binding/dissociation kinetics. Importantly, these smFRET measurements only detect FRET transitions from the high to low FRET states when nucleosome unwrapping is followed by Gal4 binding. This is because the nucleosome rewrapping rate occurs on the 10 millisecond time scale (15,39), which is much faster than our data acquisition rate. So, partially unwrapped nucleosome states not associated with Gal4 binding or dissociation are much too short lived to be detected. Furthermore, smFRET measurements only detect low to high FRET transitions for nucleosome rewrapping preceded by Gal4 dissociation. Depending on the Gal4 concentration, it is possible for Gal4 to dissociate and rebind before the nucleosome rewraps. Therefore, there is not necessarily a 1 to 1 correlation between nucleosome unwrapping/rewrapping and Gal4 binding/dissociation, respectively. This becomes important for the smFRET measurements of P31 and P36 nucleosomes.

To make progress on interpreting these complex FRET transitions, we considered 2 states for the analysis, where the high FRET states are fully wrapped nucleosomes without Gal4 bound and the low FRET states are partially unwrapped nucleosomes with Gal4 bound to its target site (Figure 5C, Supplementary Figure 5A). From these time traces, we determined the bound and unbound dwell times of each state. We then plotted the dwell times as cumulative sums and fit them to exponentials to determine the FRET transition rates (Supplementary Figure S4B). We used both single and double exponential fits and found that the data best follow a double exponential distribution for both *k*_H__→__L_ (high FRET to low FRET transition rate) and *k*_L__→__H_ (low FRET to high FRET transition rate) (Supplementary Figure S4C). This result indicates there are two distinct populations of transition rates. The primary rates are about 5 to 10-fold faster than the secondary rates for both *k*_H__→__L_ and *k*_L__→__H_. Interesting, the *K*_D_ from the ratio of the primary rates is similar to the *K*_D_ from ratio of the secondary rates, which implies that both the primary and secondary set of rates represent binding and dissociation of a single Gal4. Furthermore, this is consistent with our ensemble measurements, which indicate there is only a single concentration range of Gal4 that traps P26, P31 and P36 nucleosome unwrapping. We speculate these two separate populations of FRET transition rates are related to (i) variations in orientation of bound Gal4 relative to the histone octamer that avoids steric clash, and (ii) small changes in position of the Gal4 binding site that could occur because of changes in the histone octamer position (20) and changes in the stretching of nucleosomal DNA (35). However, since most of the FRET transitions occurred at the fast rate (Supplementary Figure S5A) for all three nucleosome samples (P26, P31 and P36) at nearly all Gal4 concentrations, we focused on the faster rates.

As previously reported, we found that 1–10 nM Gal4 induces a significant number of FRET fluctuations for P26 nucleosomes, which is consistent with the ensemble *S*_1/2_ measurements (Figure 2F). At this Gal4 binding site position, the high to low FRET transition rate, *k*_H__→__L_, increases nearly linearly implying a Gal4 effective binding rate constant of 0.08 ± 0.01 s^−1^ nM^−1^, while low to high FRET transition rate, *k*_L__→__H_, remains essentially constant implying a Gal4 dissociation rate of 0.57 ± 0.03 s^−1^ (Figure 5D, dashed lines). This result is consistent with previous measurements of Gal4 binding P26 nucleosomes (40). We observe FRET fluctuations with P31 and P36 nucleosomes in a Gal4 concentration range of 50–500 nM, a 50-fold higher concentration of Gal4 than what was required for the P26 nucleosomes, which is expected based on the ensemble *S*_1/2_ measurements (Figure 5C, E). However, the concentration dependence of *k*_H__→__L_ and *k*_L__→__H_ for P31 and P36 nucleosomes are very different from P26 nucleosomes. *k*_H__→__L_ saturates at about 0.5 s^−1^, while *k*_L__→__H_ rate decreases significantly. This implies that for P31 and P36 nucleosomes the Gal4 concentration dependence of *k*_H__→__L_ and *k*_L__→__H_ cannot be fit to a linearly increasing function and a constant value, respectively.

To understand the concentration dependence, we used the Widom site exposure model (Figure 5A) that contain 4 rates (*k*_12_, *k*_21_, *k*_23_ and *k*_32_) and includes the regime where the Gal4 binding rate can exceed the nucleosome unwrapping and rewrapping rates (see the methods section for details). To provide a tractable model of how the FRET transition rates depend on the Gal4 concentration, we included the assumption that the intermediate state (nucleosome unwrapped without Gal4 bound) is not significantly occupied. This is a reasonable approximation because the nucleosome rewrapping rate is always much larger than the unwrapping rate (15), and has been successfully used for previous experiments that use restriction enzymes to infer the accessibility of their sites within nucleosomes (12). The full functional dependence of the high to low FRET transition rate, *k*_*H*__→__*L*_, depends on the Gal4 concentration as ${k_{H \to L}} = \frac{{{k_{12}}}}{{( {1 + {k_{21}}/( {{k_{23}}[ {Gal4} ]} )} )}}\ = \frac{{{k_{12}}}}{{( {1 + {k_{12}}/( {{k_{on\ eff}}[ {Gal4} ]} )} )}}$, where (*k*_12_*/k*_21_)*k*_23_ = *k_on eff_*. The low to high FRET transition rate, *k*_*L*__→__*H*_, depends on the Gal4 concentration as ${k_{L \to H}} = \frac{{{k_{32}}}}{{( {1 + {k_{23}}[ {Gal4} ]/( {{k_{21}}} )} )}}\ = \frac{{{k_{32}}}}{{( {1 + {k_{on\ eff}}[ {Gal4} ]/( {{k_{12}}} )} )}}$. This model includes the low and high Gal4 concentration regimes. At low Gal4 concentrations, the FRET fluctuations are direct readouts of Gal4 binding and dissociation events, where *k*_*H*__→__*L*_ increases linearly as *k_on eff_* [Gal4], and *k*_*L*__→__*H*_ is a constant value of *k*_32_. In the high Gal4 concentration regime, the FRET transition is no longer a direct read out of Gal4 binding and dissociation events. Instead, the Gal4 binding rate exceeds the nucleosome rewrapping rate, so *k*_*H*__→__*L*_ is limited to the nucleosome unwrapping rate, *k*_12_. Furthermore, Gal4 exchanges multiple times before the nucleosome rewraps where *k_L_*_→__*H*_ depends hyperbolically on [Gal4] as $\frac{{{k_{21}}}}{{{k_{23}}[ {Gal4} ]}}{k_{32}}$.

By globally fitting the Gal4 concentration dependence of *k*_*H*__→__*L*_ and *k*_*L*__→__*H*_ separately for each nucleosome (P26, P31 and P36), we determined the nucleosome unwrapping rate, *k*_12_, Gal4 dissociation rate, *k*_32_, and the effective binding rate to the nucleosome, *k_on eff_* = (*k*_12_/*k*_21_) *k*_23_ (Figure 5D, Supplementary Table S2, P26: *k*_12_ = 2.5 ± 0.5 s^−1^, *k*_32_ = 0.66 ± 0.03 s^−1^, *k_on eff_* = 0.11 ± 0.03 nM^−1^ s^−1^; P31: *k*_12_ = 0.9 ± 0.1 s^−1^, *k*_32_ = 0.83 ± 0.07 s^−1^, *k_on eff_* = 0.0008 ± 0.0002 nM^−1^ s^−1^; P36: *k*_12_ = 0.9 ± 0.1 s^−1^, *k*_32_ = 1.03 ± 0.08 s^−1^, *k_on eff_* = 0.004 ± 0.001 nM^−1^ s^−1^). Note that *k_on eff_* depends on *k*_12_, which we determine independently, and the ratio, *k*_23_/*k_21_*. So, we are not able to determine *k*_23_, the Gal4 binding rate to the partially unwrapped nucleosome, nor *k*_21_, the nucleosome rewrapping rate. We find that the dissociation rate, *k*_32_, and the effective binding rate constant, *k_on eff_*, for P26 nucleosomes are similar to the simple linear model presented above. In addition, we find that for P31 and P36 nucleosomes *k_on eff_* is reduced by 130 ± 60-fold and 30 ± 10-fold, respectively, while the dissociation rate, *k*_32_, is modestly increased by 1.3 ± 0.1 -fold and 1.6 ± 0.1-fold, respectively. Importantly, the ratio of *k*_32_ and *k_on eff_* determine an effective dissociation constant that is similar to the *S*_1/2_ that were determined by ensemble FRET measurements (Figure 5E). This shows that the single-molecule measurements are consistent with the ensemble measures and indicates that the surface tethering does not influence the binding/dissociation equilibrium. Overall, these results imply that as the Gal4 binding site extends more than 30 base pairs into the nucleosome both the binding and dissociation rates changes contribute to the sudden reduction in Gal4 occupancy. However, it is the change in the binding rate that largely contributes to the abrupt 2 orders of magnitude reduction in Gal4 occupancy.

### The position dependence of the Gal4 apparent dissociation constants quantitatively agrees with the previously determined nucleosome unwrapping free energy landscape.

To fully describe nucleosome partial unwrapping, the nucleosome unwrapping free energy landscape needs to be determined (24). Previously, single-molecule force spectroscopy was used to map nucleosome unwrapping of the Widom 601 nucleosome positioning sequence at nearly base pair resolution (23). Based on this data, a nucleosome unwrapping free energy landscape has been reported for the Widom 601 nucleosome positioning sequence (24). In addition, a second force spectroscopy study reported a similar unwrapping free energy landscape (41). The Gal4 *S*_1/2_ ensemble measurements presented here for binding to its site at different positions within the nucleosome can be compared to this free energy landscape. However, there are key observations that are important to clarify before making a direct comparison. (i) The ensemble *S*_1/2_ measurements can be interpreted as the apparent dissociation constants for each binding site location. So, the relative binding free energy between binding at two different sites within the nucleosome can be related to the ensemble *S*_1/2_ measurements by ΔΔ*G*_occupancy_ = *k*_B_*T* ln (*S*_1/2 position_/*S*_1/2 reference_). (ii) Our finding that it is largely the Gal4 binding rate that is reduced as the binding site is moved further into the nucleosome implies that the ΔΔ*G*_occupancy_ should be approximately equal to the ΔΔ*G*_unwrap_. (iii) Our finding that the nucleosomal DNA does not unwrap significantly past its binding site indicates that the ΔΔ*G*_unwrap_ for *x* base pairs unwrapped should be compared to the ΔΔ*G*_occupancy_ where the inner side of the Gal4 binding site is within a few base pairs of *x*.

**Figure 6.**
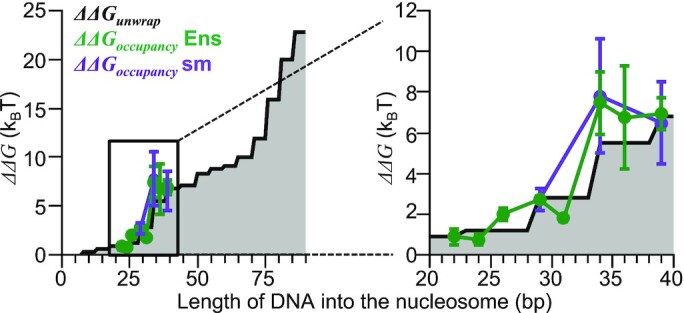
Relative free energy landscape of Gal4 binding closely matches the nucleosome unwrapping landscape. Gray area plot represents the calculated free energy landscape of nucleosome unwrapping (24). Green and purple lines represent the relative free energy of Gal4 binding from ensemble and single-molecule experiments, respectively. The binding measurements were mapped onto the DNA base pairs into the nucleosome axis by assuming the DNA unwraps 3 bp beyond the inner side of the Gal4 target sequence. Both *ΔΔG* landscapes of Gal4 occupancy are calibrated to position 29 of the unwrapping landscape. Error bars indicate ±1 SD.

Based on these observations, we compared the ΔΔ*G*_occupancy_ that is inferred from the ensemble S_1/2_ measurements as a function of the distance the Gal4 binding site extends into the nucleosome to the previously published 601 nucleosome unwrapping free energy landscape, ΔΔ*G*_unwrap_ (Figure 6). If the nucleosome is allowed to unwrap 3 bp beyond the TF binding site at each position, we find that the Gal4 binding free energy differences is not statistically different from the published nucleosome unwrapping free energy landscape as determined by the Kolmogorov–Smirnov test (*P* = 0.093). In addition, we compared the relative change in ΔΔ*G*_occupancy_ as determined from the single-molecule kinetic measurements of nucleosome unwrapping and rewrapping (Supplementary Table S2), which are also consistent with the nucleosome unwrapping free energy landscape (Figure 6). This agreement strongly indicates that the nucleosome unwrapping free energy landscape is the primary determinant of Gal4 occupancy at different positions within the nucleosome, while additional factors including the binding site orientation and the accelerated Gal4 dissociation rate appear to be at most minor contributors to the binding site position dependence of Gal4 occupancy.

## DISCUSSION

In this study, we used ensemble and single-molecule fluorescence approaches to determine the impact of binding site position on the binding equilibria and kinetics of both Gal4 binding and dissociation, and partial nucleosome unwrapping and rewrapping. We found that the positional dependence of Gal4 occupancy is dominated by the nucleosome unwrapping free energy landscape. In addition, the steric bulk of Gal4 did not require the DNA to unwrap significantly past the binding site. Importantly, we find that the nucleosome must unwrap, so Gal4 binds to the full site when both CCG sequences are present. Gal4 contains small zinc finger domains that recognize the three base sequence CCG situated at both 5 prime ends of the binding site, while the dimerization domain extends out from the middle of the binding site (28). This suggests that the low-profile zinc fingers of Gal4 facilitate nucleosome invasion by minimizing the amount of DNA that needs to be unwrapped from the nucleosome.

An important observation of this study is the sudden increase in free energy for Gal4 to occupy its site within a partially unwrapped nucleosome as the binding site position is shifted from extending 29 bp to 34 bp into the nucleosome. This increase in the free energy follows the unwrapping free energy landscape quantitatively (Figure 6), which is the key support for the conclusion that the unwrapping free energy landscape is the primary determinant of the position dependence of Gal4 occupancy within the nucleosome. This rapid free energy increase can be viewed as a well-defined barrier to TF occupancy where the probability for Gal4 occupancy decreases by over 100-fold implying that a 100-fold higher concentration of Gal4 is required for efficient binding within the nucleosome. For Gal4, the concentration required for nucleosome binding shifted from a few nanomolar to hundreds of nanomolar. This is in the physiological range of known TF concentrations (42) indicating both regions could be accessible to TFs via site exposure. However, the presence of competitor non-specific DNA sites complicates the estimate of free Gal4 concentration. Finally, the TF concentration will have an abrupt instead of gradual influence on the distance into the nucleosome that is accessible to TF occupancy.

These Gal4 binding studies at sites nearest the nucleosome edge (P19, P21, P23) provide insight into the complex nature of TF binding within partially unwrapped nucleosomes. Initial Gal4 binding induces a small change in FRET, the binding curves extend four orders of magnitude, and exhibit two distinct ‘turn-overs.’ The first Gal4 binding to partially unwrapped P19 nucleosomes has a reduction in the FRET efficiency (0.17 ± 0.01) that is significantly less than P21 (0.36 ± 0.01) and P23 (0.30 ± 0.01) (Supplementary Figure S1). This suggests that the amount of DNA unwrapped for the first Gal4 to bind is less in P19 nucleosomes than for P21 or P23 nucleosomes, which is consistent with the relative locations of the Gal4 site within the nucleosome. Interestingly, the FRET reduction of P21 is similar but slightly larger than with P23, suggesting that P21 nucleosomes have a similar but slightly larger amount of unwrapped DNA than P23 when bound with one Gal4. These results indicate that while the FRET reduction from the first Gal4 binding event is qualitatively consistent with amount of DNA unwrapped, the magnitude of the associated FRET reduction likely depends on additional factors, such as the unwrapped DNA bending out of plane to accommodate steric clash between Gal4 and the nucleosome.

The free energy landscape can help explain two additional observations from the ensemble FRET measurements. The first observation is that the binding of the second Gal4 to P19, P21 and P23 nucleosomes induces a smaller FRET change (implying less additional nucleosome unwrapping) as the binding site is moved more internally (Supplementary Figure S1). This is contrary to the expectation that as the target site is positioned further into the nucleosome the second binding requires DNA to unwrap further into the nucleosome resulting in a large FRET reduction. For P19–P23 nucleosomes, <30 bp need to unwrap for the first Gal4 to bind. The free energy landscape likely restricts the additional DNA unwrapping to a total of about 30 base pairs into the nucleosome. So, as the second Gal4 binds, less DNA unwraps as the site is moved from position P19 to P23. The second observation is that we only observe one Gal4 bind P26-P36 nucleosomes. Gal4 binding to P26-P36 nucleosomes requires 30 bp or more to unwrap. The much higher free energy cost of nucleosome unwrapping past 30 bp could prevent the addition DNA unwrapping for a second Gal4 to being within these nucleosomes (P26–P36). However, unlike P19–P23 nucleosomes, initial Gal4 binding at P26–P36 produces a large initial change in FRET efficiency, perhaps rendering the FRET measurements of these nucleosomes insensitive to additional unwrapping, which prevents detection of binding of a second Gal4.

In these studies, we used the high affinity Widom 601 NPS. The stability of this sequence raises the question of whether the well-defined barrier to TF occupancy for binding sites that extend >30 bp into the nucleosome is largely due to this NPS, which was selected for nucleosome stability. While this is possible, there are multiple observations that indicate that the reported shape of the 601 NPS landscape will be similar for other DNA sequences out to 36 bp into the nucleosome, the region of nucleosomal DNA studies here. (i) the 601 NPS was selected for DNA interactions with the H3-H4 tetramer (43). So, the 601 NPS was not selected for interactions between the DNA 30 and 35 bp into the nucleosome and the H2A–H2B heterodimer. (ii) DNA interactions with the H2A–H2B dimer around 30 bp into the nucleosome on the opposite (right) side of the 601 NPS is measurably weaker based on previous force spectroscopy measurements (23) and the preferential binding of H2A-H2B heterodimers to the left side of 601 NPS. However, these changes are due to free energy change on the *k*_B_*T* scale. So, the sudden large increase in the unwrapping free energy landscape does not vary much between the two sides of the 601 NPS (25). (iii) Finally, despite inserting the 19 bp Gal4 binding site into the 601 NPS at 8 different positions, which significantly changes the 601 NPS sequence, the change in binding free energy matched the unwrapping free energy landscape for the original 601 NPS that does not contain the Gal4 target sequence. These combined observations support the conclusion that the overall unwrapping free energy landscape will not change significantly between DNA sequences and that the sudden rise in unwrapping free energy between 30 and 35 bp into the nucleosome will remain for most DNA sequences.

**Figure 7.**
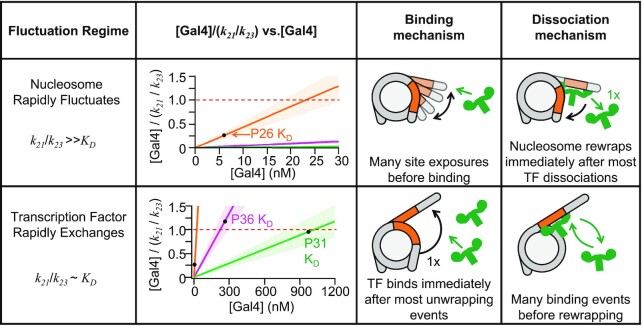
Two modes of TF occupancy within a nucleosome entry–exit region. Top row: For binding sites contained within the first 30 bp of the nucleosome, *k*_21_/*k*_23_ is much larger than the dissociation constant, *K*_D_. For P26 nucleosomes, the *K*_D_ value on a plot of [Gal4]/(*k*_21_/*k*_23_) versus [Gal4] (orange line) is significantly below 1, which is the Gal4 concentration that equals *k*_21_/*k*_23_ (red dashed line). As Gal4 significantly occupies its binding site at concentrations around the *K*_D_, (i) the binding mechanism continues to involve the nucleosome undergoing many unwrapping/rewrapping fluctuations before a Gal4 binding event traps the nucleosome in an unwrapped state, and (ii) the dissociation mechanism results in most Gal4 dissociation events being followed by nucleosome rewrapping. Bottom row: For binding sites that extend >30 bp into the nucleosome, *k*_21_/*k*_23_ is similar to the dissociation constant, *K*_D_. For P31 and P36 nucleosomes, the *K*_D_ value on a plot of [Gal4]/(*k*_21_/*k*_23_) versus [Gal4] (green and magenta lines, respectively) is close to 1, which is the Gal4 concentration that equals *k*_21_/*k*_23_ (red dashed line). As Gal4 significantly occupies its binding site at concentrations around the *K*_D_, (i) the binding mechanism is limited by the nucleosome unwrapping rate, so Gal4 binds its site after each unwrapping event, and (ii) the dissociation mechanism results in multiple Gal4 dissociation and rebinding events before the nucleosome unwraps. Error indicates ±1 SD from single-molecule measurement of *k*_21_/*k*_23_.

In addition to the sudden change in Gal4 occupancy at about 30–35 bp into the nucleosome, there is a qualitative change in the Gal4 concentration dependence of the nucleosome transition rates to the unwrapped state that is bound by Gal4, *k*_H__→__L_, and to the state where the nucleosome is fully wrapped and not bound by Gal4, *k*_L__→__H_ (Figure 7). For binding sites <30 bp into the nucleosome, *k*_H__→__L_ increases linearly with Gal4 concentration, while *k*_L__→__H_ is constant. This can be understood by comparing the apparent *K*_D_, which is equal to *k*_32_/*k*_23_ and represents the characteristic concentration for Gal4 occupancy, to *k*_21_/*k*_23_, which is also in units of concentration and is the Gal4 concentration at which the two transition rates out of state 2 are equal. Therefore, at Gal4 concentrations around and above *k*_21_/*k*_23_, Gal4 will dissociate and rebind multiple times before the nucleosome rewraps. At P26, the *K*_D_ is significantly less than *k*_12_/*k*_23_ (Figure 7), which implies that Gal4 occupancy largely saturates before Gal4 reaches a concentration where Gal4 dissociates and rebinds multiple times before the nucleosome rewraps. Therefore, in this outer region of the nucleosome there is essentially a one-to-one correspondence of rewrapping events to Gal4 dissociation events until the binding site is saturated.

In contrast, for binding site locations that extend >30 bp into the nucleosome, *k*_H__→__L_ converges to a constant value and *k*_L__→__H_ decreases hyperbolically as the Gal4 concentration increases even though the Gal4 concentrations are around the apparent *K*_D_ and the binding site is not fully saturated. In this region, the apparent *K*_D_ is similar to the ratio, *k*_21_/*k*_23_. Therefore, as Gal4 starts to occupy its site within the partially unwrapped nucleosomes, Gal4 is at a concentration where the nucleosome rewrapping rate (*k*_21_) becomes comparable to the Gal4 binding rate to partially unwrapped nucleosomes (*k*_23_ [Gal4]). Under these conditions the Gal4 binding rate is limited by the unwrapping rate (*k*_12_), and Gal4 dissociates-rebinds multiple times before the nucleosome can unwrap. Therefore, nucleosome rewrapping events no longer have a one-to-one correspondence with Gal4 dissociation events, while Gal4 binds every time the nucleosome unwraps (Figure 7). These observations imply that not only is the nucleosome unwrapping free energy landscape the primary determinant for the position dependence of Gal4 occupancy within nucleosomes, but as the binding site shifts further into the nucleosome, the connection between nucleosome unwrapping and Gal4 binding quantitatively shifts such that the nucleosome rewrapping is significantly slowed even before Gal4 fully occupies its binding site. Overall, these results provide important insight into how a TF can influence the nucleosome unwrapping/rewrapping kinetics, which has important ramifications on how Gal4 functions as an activator of transcription, which could contribute to transcriptional bursting.

Our observation that the orientation of the binding site relative to the histone octamer had a modest impact on Gal4 occupancy is surprising since the orientation changes significantly as the binding site is moved only a few base pairs relative to the nucleosome structure because of the ∼10 bp helical repeat of dsDNA. This implies that irrespective of orientation, the DNA unwraps to a similar position relative to the Gal4 binding site and is consistent with our FRET measurements that indicate that the DNA does not need to unwrap significantly past the inner edge of the Gal4 binding site. The Gal4-DNA crystal structure shows that the DNA is essentially straight when bound by the Gal4 dimer, which occupies 270° of the target site. Combined these observations suggest that the Gal4 site needs to unwrap completely from the histone octamer surface to straighten so that Gal4 can fully bind, and that this amount of unwrapping allows for Gal4 to bind without steric clash with the nucleosome.

We previously reported that the Gal4 dissociation rate is dramatically accelerated from its binding site within a partially unwrapped nucleosome (22). This accelerated dissociation can be explained by a competition model between states where the nucleosome is unwrapped so Gal4 is fully bound to its binding site and states where the nucleosome is less unwrapped so that only the outer portion of the Gal4 binding site is exposed and Gal4 is partially bound to its binding site (44). Previously, there has not been direct evidence for this model. However, our studies here of Gal4 occupancy at mutated Gal4 binding sites: Outer_binding_ and Inner_binding_, provides two forms of evidence in support of this competition model. (i) The *S*_1/2_ (which is also the apparent *K*_D_) of these mutated sites is significantly higher than the *S*_1/2_ for the Full_binding_ site (Figure 2). This implies that both the inner and outer CCG is important for Gal4 binding, ruling out a model where there is no competition and Gal4 only partially binds to the outer CCG. (ii) The reduction in FRET induced by Gal4 binding is always smaller for the Outer_binding_ site than the Inner_binding_ site and is particularly noticeable at positions P21 and P23. This implies that the nucleosome is less unwrapped for Gal4 binding to the Outer_binding_ site than the Inner_binding_ site. This in turn indicates that at the mutated site, when Gal4 is partially bound to the Outer_binding_ site, the inner CCG is wrapped into the nucleosome. In combination, these results support the competitive model between Gal4 binding the inner CCG and the nucleosome rewrapping the CCG.

Finally, single-molecule measurements of transcription have revealed that they occur in bursts (2). Recently, it was reported that the burst duration for transcription of the *Gal3* gene is limited by the lifetime of Gal4 bound in the promoter, which is accelerated off by the nucleosome (40). Since the accelerated dissociation is not dependent on the location of the binding site within the nucleosome, our findings suggest that the transcription burst duration will not be highly sensitive to the precise position of the Gal4 binding site within the nucleosome. This highlights how not only equilibrium occupancy of TFs at their target sites, but the length of time they reside at their site is important for the transcriptional output of the gene they are activating.

## DATA AVAILABILITY

The experimental data sets are either included in this manuscript, the supplemental information, or are available from the authors upon request. The single molecule fluorescence traces are available at Zenodo, https://doi.org/10.5281/zenodo.7464019.

## Supplementary Material

gkac1267_Supplemental_File

## Notes

Present address: Yi Luo, Illumina Inc, Product Development, 5200 Illumina Way, San Diego, CA 92122, USA.
