# Supplementary material for: The nucleosome unwrapping free energy landscape defines distinct regions of transcription factor accessibility and kinetics
Source: Nucleic Acids Res. 2023 Jan 23;51(3):1139–53. doi: 10.1093/nar/gkac1267 (PMC9943653; doi:10.1093/nar/gkac1267)
Supplement: gkac1267_Supplemental_File [file gkac1267_supplemental_file.pdf]

SUPPLEMENTAL FIGURES

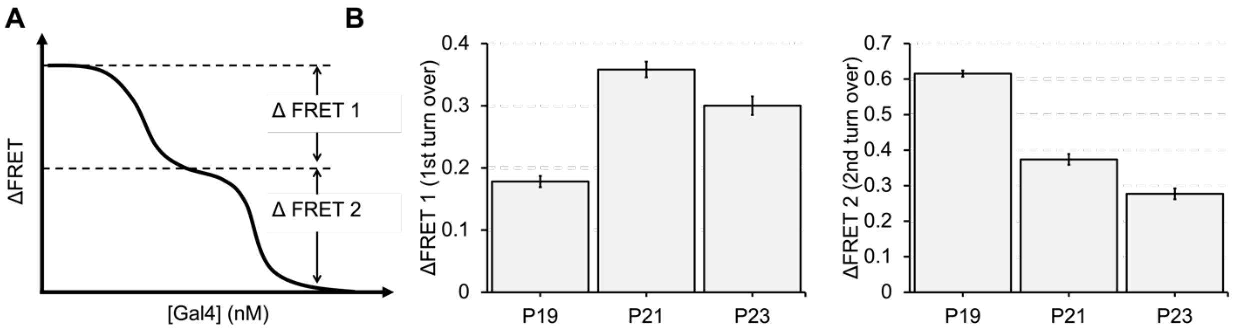

Figure S1. Magnitude of FRET change for each Gal4 binding event for P19, P21, and P23 nucleosomes. A) Cartoon illustration of bimodal binding curve defining ΔFRET 1 as the FRET reduction due to binding of one Gal4 and ΔFRET 2 as the FRET reduction upon binding a second Gal4. B) Bar plots of ΔFRET 1 and ΔFRET 2 for P19, P21, and P23 nucleosomes. Error bars indicate +/- 1 SEM.

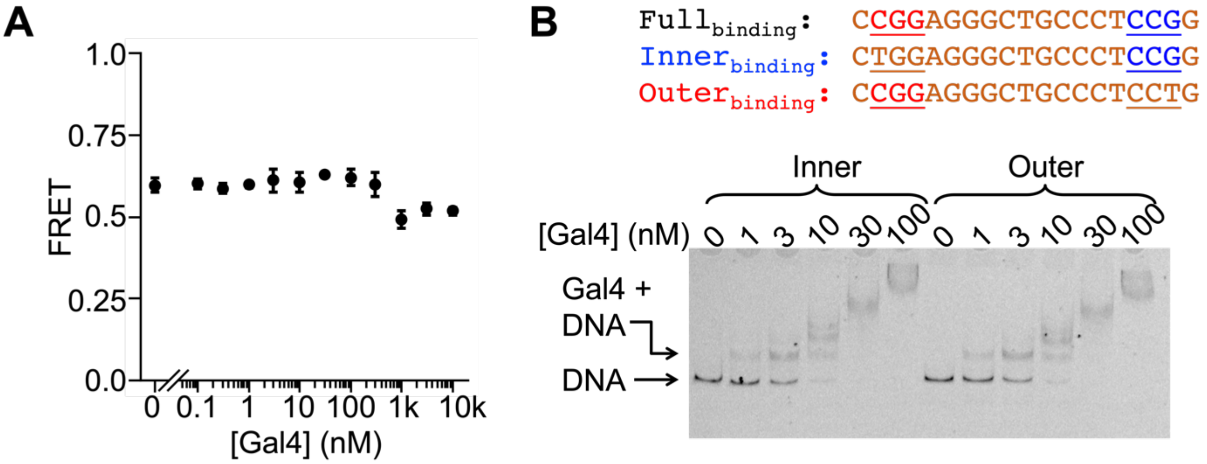

Figure S2. Measuring Gal4 site specificity within nucleosomes and DNA. (A) Titrating Gal4 while measuring FRET efficiency between H2A(K119C) and end-labeled Cy3 DNA for nucleosomes that do not contain a Gal4 binding site. This experiment shows that Gal4-induced nucleosome unwrapping requires a Gal4 binding site. Error bars indicate +/- 1 SEM. (B) Measuring Gal4 binding to 'inner' and 'outer' half site mutants on DNA. Gal4 binding affinity is detected by electrophoretic mobility shift assay (EMSA). Gal4 binds with the same affinity to both DNAs.

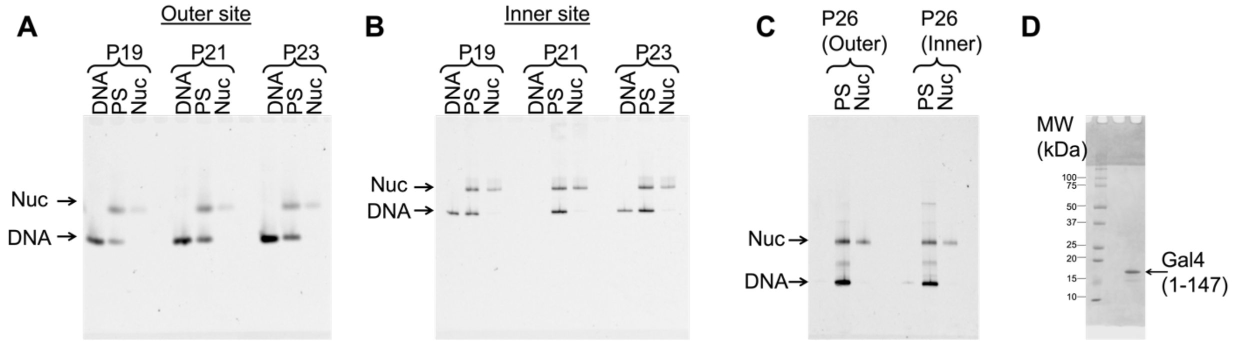

Figure S3. Nucleosomes containing mutated Gal4 binding sites used in this study. (A) Native gel of reconstituted nucleosomes containing “outer” Gal4 binding site mutations at the indicated position (P19, P21, P23). “DNA" = DNA used containing Gal4 binding site, “PS” nucleosomes post reconstitution by salt gradient dialysis but before purification by sucrose gradient, “Nuc” = final nucleosomes used in experiments. (B) Native gel of reconstituted nucleosomes containing “inner” Gal4 binding site mutations at the indicated position (P19, P21, P23). (C) Native gel of nucleosomes containing ”outer” and “inner” mutations to Gal4 binding site at position P26. (D) Purified Gal4 visualized on SDS PAGE gel.

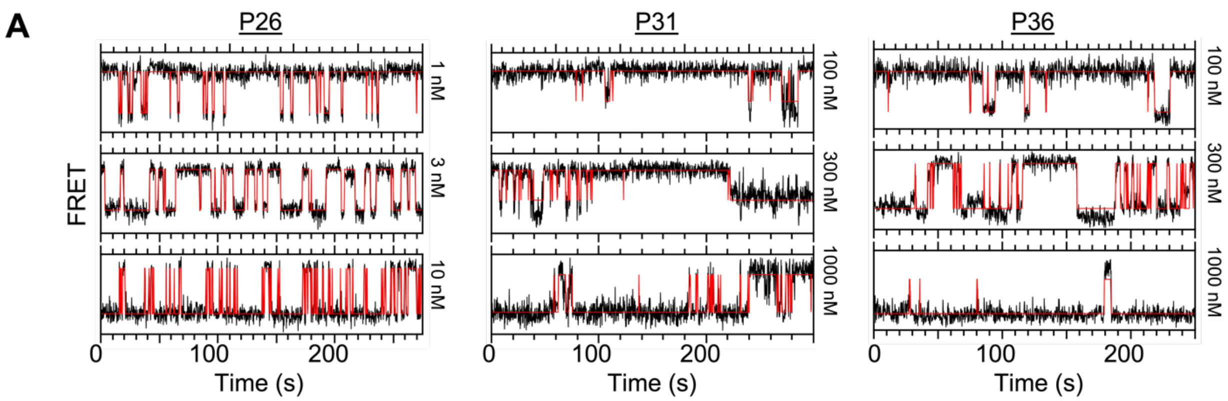

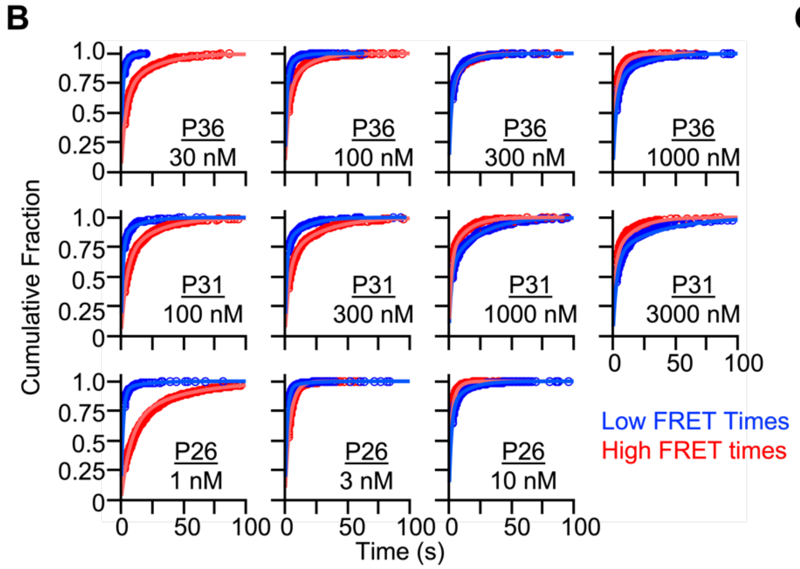

C

H_0: Distribution follows single exponential
P-value < 0.01, reject

P26	P-value k_{on}	P-value k_{off}
1	<1E-16	<1E-16
3	<1E-16	<1E-16
10	<1E-16	<1E-16
P31		
100	<1E-16	<1E-16
300	<1E-16	<1E-16
1000	<1E-16	<1E-16
3000	<1E-16	<1E-16
P36		
30	<1E-16	<1E-16
100	<1E-16	<1E-16
300	<1E-16	<1E-16
1000	<1E-16	<1E-16

Figure S4. Measuring Gal4 binding kinetics to nucleosomes by single-molecule FRET. (A) Representative single-molecule FRET time traces of Gal4 binding to P26, P31, and P36 nucleosomes at multiple Gal4 concentrations. Raw FRET data (black) is fit to a 2-state Hidden-Markov Model (red) which represents wrapped (high FRET state) and unwrapped (low FRET state) nucleosomes. (B) Cumulative distribution and associated 2-exponential fit for each single-molecule experiment in this study. These fit values were used to calculate the transition rates in figure 5D. (C) Results of likelihood ratio tests to determine whether to fit data to single- or double-exponential distribution. If p-values are below 0.01, we reject the null hypothesis that distribution follows single-exponential decay.

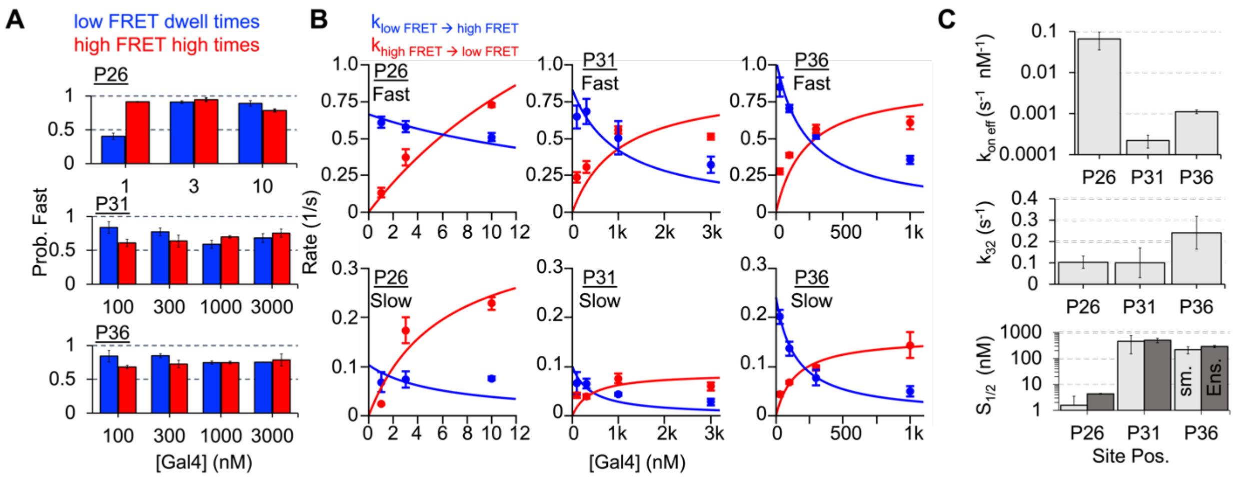

Figure S5. Analysis of Gal4 binding kinetics to nucleosomes. (A) Fraction of dwell times belonging to "fast" population determined from double-exponential fit for both low FRET (blue) and high FRET (red) dwell times. (B) Determination of High--> low transition rate (red) and low --> high transition rate (blue) for slow population of Gal4 binding to P26, P31, and P36 nucleosomes. Data is fit to 3 state binding model depicted in figure 5A. Error bars indicate +/- 1 SD. (C) Summary of slow population of rates: effective k_{on} (top panel), the Gal4 dissociation rate from nucleosomes (k_{32}) (middle panel), and $S_{1/2}$ values from both single-molecule FRET and ensemble FRET measurements (bottom panel). Error bars indicate +/- 1 SD.

SUPPLEMENTAL TABLES

Primer	sequence
Gal42C_P19_fwd	Cy3-CCGGAGGGCTGCCCTCCGGAGGCCGCTCAATTGGTCGTAGCAAGCTCTAGCACCGC
Gal42C_P21_fwd	Cy3-CTCCGGAGGGCTGCCCTCCGGGCCGCTCAATTGGTCGTAGCAAGCTCTAGCACCGC
Gal42C_P23_fwd	Cy3-CTGGCCGGAGGGCTGCCCTCCGGCGCTCAATTGGTCGTAGCAAGCTCTAGCACCGC
Gal42C_P26_fwd	Cy3-CTGGAGACCGGAGGGCTGCCCTCCGGTCAATTGGTCGTAGACAGCTCTAGCACCGC
Gal42C_P28_fwd	Cy3-CTGGAGAATCCGGAGGGCTGCCCTCCGGAATTGGTCGTAGCAAGCTCTAGCACCGC
Gal42C_P31_fwd	Cy3-CTGGAGAATCCCCCGGAGGGCTGCCCTCCGGTGGTCGTAGCAAGCTCTAGCACCGC
Gal42C_P33_fwd	Cy3-CTGGAGAATCCCGGCCGGAGGGCTGCCCTCCGGGTCGTAGCAAGCTCTAGCACCGC
Gal42C_P36_fwd	Cy3-CTGGAGAATCCCGGTGCCCGGAGGGCTGCCCTCCGGGTAGCAAGCTCTAGCACCGC
No site_fwdN	Cy3-CTGGAGAATCCCGGTGCCGAGGCCGCTCAA
Rvs for sm (222 bp)	Biotin-CGCATGCTGCAGACGCGTTACGTATCGGATCC
Rvs for ens (147 bp)	ACAGGATGTATATATCTGACACGTGCCTGG
D17-fwd	CTGGAGACCGGAGGGC[aminoC6dT]GCCCTCCGGTCAATTGGTCGTAGACAGCTCTAGCACCGC
D27-fwd	CTGGAGACCGGAGGGCTGCCCTCCGG[aminoC6dT]CAATTGGTCGTAGACAGCTCTAGCACCGC
P19_Outer	Cy3-C**TGG**AGGGCTGCCCT**CCG**GAGGCCGCTCAATTGGTCG
P21_Outer	Cy3-CTC**TGG**AGGGCTGCCCT**CCG**GGCCGCTCAATTGGTCG
P23_Outer	Cy3-CTGGC**TGG**AGGGCTGCCCT**CCG**GCGCTCAATTGGTCG
P26_Outer	Cy3-CTGGAGAC**TGG**AGGGCTGCCCT**CCG**GTCAATTGGTCG
P28_Outer	Cy3-CTGGAGAATC**TGG**AGGGCTGCCCT**CCG**GAATTGGTCG
P19_Inner	Cy3-C**CGG**AGGGCTGCCCT**CCT**GAGGCCGCTCAATTGGTCG
P21_Inner	Cy3-CTC**CGG**AGGGCTGCCCT**CCT**GGCCGCTCAATTGGTCG
P23_Inner	Cy3-CTGGC**CGG**AGGGCTGCCCT**CCT**GCGCTCAATTGGTCG
P26_Inner	Cy3-CTGGAGAC**CGG**AGGGCTGCCCT**CCT**GTCAATTGGTCG
P28_Inner	Cy3-CTGGAGAATC**CGG**AGGGCTGCCCT**CCT**GAATTGGTCG

Table S1. List of oligos used in this study. The green boxes indicate the Gal4 binding site. The red letters indicate the base pair substitution to asymmetrically weaken the Gal4 binding affinity.

Position	k_{12} (sec^{-1})	k_{32} (sec^{-1})	k_{21}/k_{23} (nM)	$k_{on\ eff}$ = $(k_{12}/k_{21})k_{23}$ (nM^{-1} sec^{-1})	K_D = $(k_{32}/k_{23})/(k_{12}/k_{21})$ (nM)	$\Delta\Delta G_{binding}$ = $-k_BT \ln(K_D/K_{D\ P26})$ (k_BT)
P26	2.5 ± 0.5	0.66 ± 0.03	23 ± 6	0.11 ± 0.03	6.1 ± 1.9	0
P31	0.9 ± 0.1	0.83 ± 0.07	1020 ± 280	0.0008 ± 0.0002	980 ± 310	5.1 ± 2.3
P36	0.9 ± 0.1	1.03 ± 0.08	220 ± 50	0.004 ± 0.001	260 ± 70	3.7 ±1.5

Table S2. Fit values from single-molecule experiments. Error indicate +/- 1 SD.

	P26				P31				P36			
	fast rate		slow rate		fast rate		slow rate		fast rate		slow rate	
	rate	err	rate	err	rate	err	rate	err	rate	err	rate	err
k_{12}	2.525	0.472	0.388	0.078	0.869	0.110	0.087	0.010	0.879	0.083	0.160	0.006
k_{32}	0.664	0.029	0.103	0.020	0.832	0.070	0.101	0.013	1.035	0.077	0.241	0.009
$k_{on\ eff}$	0.11	0.03	0.07	0.03	0.0008	0.0002	0.0002	0.0001	0.004	0.001	0.0011	0.0001

Table S3. Fit values for fast and slow populations of binding. Error bars indicate +/- 1 SD.
